# Central Pain in Parkinson's Disease: Behavioral and Cognitive Characteristics

**DOI:** 10.1155/2021/5553460

**Published:** 2021-06-10

**Authors:** N. Vila-Chã, S. Cavaco, A. Mendes, A. Gonçalves, I. Moreira, J. Fernandes, J. Damásio, L. F. Azevedo, J. M. Castro-Lopes

**Affiliations:** ^1^Department of Neurology, Centro Hospitalar Universitário do Porto, Porto, Portugal; ^2^Laboratory of Neurobiology of Human Behavior, Centro Hospitalar Universitário do Porto, Porto, Portugal; ^3^Unity in Multidisciplinary Research on Biomedicine (UMIB), Abel Salazar Biomedical Sciences Institute, University of Porto, Porto, Portugal; ^4^Centre for Health Technology and Services Research (CINTESIS), University of Porto, Porto, Portugal; ^5^Institute for Molecular and Cell Biology (IBMC), University of Porto, Porto, Portugal; ^6^National Observatory for Pain–NOPain, Faculty of Medicine, University of Porto, Porto, Portugal; ^7^Department of Community Medicine, Information and Health Decision Sciences (MEDCIDS), Faculty of Medicine, University of Porto, Porto, Portugal; ^8^Department of Biomedicine, Faculty of Medicine, University of Porto, Porto, Portugal

## Abstract

**Introduction:**

Pain is a major nonmotor symptom of Parkinson's disease (PD), and central parkinsonian pain is the core feature of the putative Park pain subtype of PD. This study aimed to explore the cognitive and behavioral profile of PD patients with central parkinsonian pain. *Material and Methods*. A structured interview was used to identify and characterize pain in a cohort of 260 consecutive PD patients. The Ford classification of pain was applied. The Dementia Rating Scale-2 (DRS-2) and the Impulse Control Disorders in Parkinson's Disease Short Form (QUIP-S) were administered, and patients' smoking habits were recorded. The Unified Parkinson's Disease Rating Scale (UPDRS) was used to assess motor and nonmotor symptoms in *off* and *on* conditions.

**Results:**

One hundred and eighty-eight patients (68%) reported pain; and in 41 (22%) of them, the pain was classified as central parkinsonian pain. PD patients with central parkinsonian pain had better cognitive performance in DRS-2 Initiation/Perseveration and Conceptualization subscales but reported more other compulsive behaviors (e.g., hobbyism, punding, and walkabout) and had more current smoking habits than those without pain or with non-central parkinsonian pain. Multiple logistic regression analyses revealed that the DRS-2 Conceptualization subscale, other compulsive behaviors, and smoking habits remained statistically associated with central parkinsonian pain even when other significant covariates were considered. Only patients with pain, regardless of type, had a gambling disorder. *Discussion*. The study results provide further evidence that pain revealed that patients with central parkinsonian pain are more likely to present compulsive or addictive behaviors, despite having more preserved cognitive performance. Patients with central parkinsonian pain appear to have a distinct phenotype of PD.

## 1. Introduction

Parkinson's disease (PD) is a complex neurodegenerative disorder that includes motor and nonmotor symptoms, such as dementia, sleep disorders, autonomic dysfunction, sensory, and psychiatric symptoms [1]. It has been recognized that PD is highly heterogeneous, regarding clinical presentation and progression [2]. The emergence of patterns of co-occurrence or clustering of certain nonmotor symptoms has led to the proposal of nonmotor subtypes of PD [3, 4]. Sauerbier et al. has suggested seven specific nonmotor symptom-dominant phenotypes, including a Park pain subtype [3]. It has been speculated that these different subtypes may have distinct pathogenesis [4].

Pain in PD is a major nonmotor symptom with a prevalence of up to 85% and is associated with poorer quality of life [5]. Central parkinsonian pain is believed to be the only subtype of pain that is directly related to the disease itself and is the core feature of the putative Park pain subtype [6]. Patients with central parkinsonian pain are known to be younger, have earlier disease onset, fewer comorbidities, greater nonaxial motor symptom severity in *on*, more pain‐related disability, more sleep disturbances, and more relief of pain with antiparkinsonian medication than patients with non‐central parkinsonian pain [7, 8]. It is also widely recognized that younger patients with PD usually have more preserved cognition and are at a higher risk of presenting impulse control disorders [9, 10].

The general aim of this study was to carry out a cognitive and behavioral characterization of PD patients with pain, specifically those with central parkinsonian pain.

## 2. Material and Methods

### 2.1. Participants

A cross-sectional study of PD patients was carried out in the Movement Disorders Clinic of Centro Hospitalar Universitário do Porto (CHUPorto). Full details of the protocol have been described in a previous article [7]. In brief, patients were eligible for inclusion if they met the United Kingdom Brain Bank criteria for diagnosis of PD [11]. Drug-induced parkinsonism, possible or probable atypical parkinsonian syndromes, vascular parkinsonism, and advanced therapies (i.e., subcutaneous apomorphine pump, levodopa-carbidopa intestinal gel, or deep brain stimulation) were considered a priori exclusion criteria.

**Figure 1:**
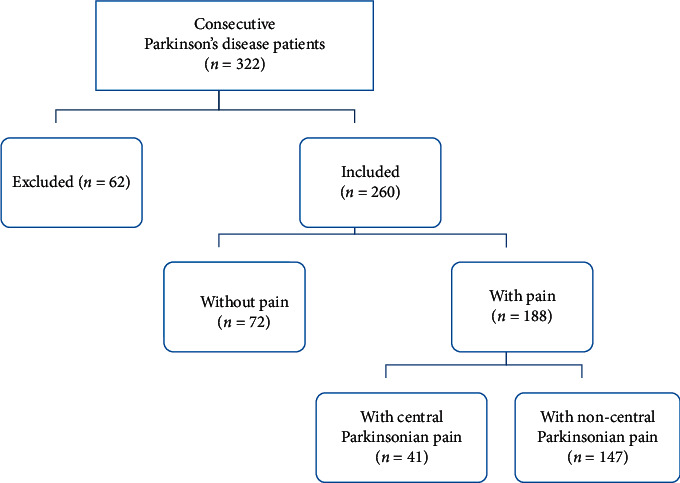
Flowchart of the study sample.

From a consecutive series of 322 possible subjects, 260 participated in the study (Figure 1). One patient refused to participate in the study, 53 were excluded before assessment (i.e., 13 moved geographically to a region not dependent from our center or could not be reached between inclusion and assessment, 4 could not be assessed due to logistic problems, 2 developed other debilitating conditions, 7 died, and 27 had less than three years of education) and 8 were excluded after the assessment (i.e., 3 due to inability to complete the Dementia Rating Scale-2 (DRS-2), 3 had a change in the diagnosis, and 2 due to incomplete data set).

All the patients (or legal representatives) were informed about the nature of the study and gave their written informed consent. The ethics committee of CHUPorto approved the study.

### 2.2. Procedures

A movement disorders specialist performed a semistructured interview (Supplementary Material (available here)) and a neurological examination to all participants. PD patients were evaluated in the morning without antiparkinsonian medication for 12 hours (*off* medication condition) using the Unified Parkinson's Disease Rating Scale-III (UPDRS-III) [12]. After the assessment in *off* condition, patients took their usual first dose of antiparkinsonian medication and were re-evaluated one hour later (*on* medication condition) using the same instruments. The UPDRS subscale for activities of daily living (UPDRS-II) was also applied regarding *off* and *on*. Levodopa responsiveness was calculated as the percent change in UPDRS score [i.e., (OFF − ON)/OFF *∗* 100]. All patients were asked whether they had pain in the last month. Those who responded “yes” to the previous question were asked a series of questions regarding their pain. Based on the patients' description, the neurologists categorized the pain, according to the Ford framework [6], as central parkinsonian pain, musculoskeletal pain, dystonia-related pain, radicular or neuropathic pain, and/or akathitic discomfort. Central parkinsonian pain was defined, according to the Ford criteria [6], as burning, tingling, formication, or “neuropathic” sensations, often relentless and bizarre in quality, not confined to root or nerve territory, and not explained by rigidity, dystonia, musculoskeletal, or internal lesion. The neurologists used the Questionnaire for Impulse Control Disorders in Parkinson's Disease Short Form (QUIP-S) to identify participants with impulse control disorders (ICDs; i.e., gambling, sexual, buying, and eating behaviors), other compulsive behaviors (i.e., hobbyism, punding, and walkabout), and compulsive medication use [13, 14]. Patients' past and current smoking habits were recorded.

A trained neuropsychologist applied the Portuguese version of the DRS-2 [15]. Test scores were adjusted for demographic characteristics (i.e., age and education) according to the national norms, and the fifth percentile of the norms was used as cutoff for cognitive impairment. DRS-2 was applied under the effect of the regular antiparkinsonian medication (in *on* condition).

### 2.3. Statistical Analysis

Descriptive statistics were used for group characterization and nonparametric tests (i.e., chi-square, Fisher's exact, and Mann–Whitney) were applied for group comparisons. The threshold for statistical significance for group comparisons was *p* < 0.05. Multiple logistic regressions explored group differences while considering relevant covariates. The backward selection method was applied with a threshold for variable removal of *p* > 0.100. The statistical analysis was conducted using the Statistical Package for the Social Sciences, version 25.0 (SPSS, USA).

## 3. Results

### 3.1. Total Sample

Of the 260 PD patients who were examined, 135 (52%) were men and 125 (48%) were women, with median age = 70 years, education = 4, age at disease onset = 60 years, and disease duration = 7 years. At the time of the assessment, 188 patients (68%) reported pain. Of those with pain, 41 (22%) had central parkinsonian pain and 147 (78%) had non-central parkinsonian pain. The demographic and clinical characteristics of the subgroups are presented in Table 1.

### 3.2. Central Parkinsonian Pain

As compared with patients without pain and patients with non-central parkinsonian pain, PD patients with central parkinsonian pain were younger at disease onset (*p*=0.002 and *p*=0.011, respectively) and at assessment (*p*=0.001 and *p*=0.002 respectively) and were taking more dopamine agonists (*p*=0.031 and *p*=0.024, respectively) (Table 1). Patients with central and non-central parkinsonian pain had a higher UPDRS-II score in the *off state* than patients without pain (*p*=0.001 and *p*=0.002, respectively). PD patients with central parkinsonian pain had greater UPDRS-II levodopa responsiveness than patients with non-central parkinsonian pain (*p*=0.001), and the responsiveness was higher than patients without pain although the level of statistical significance was marginal and nonsignificant (*p*=0.063).

PD patients with central parkinsonian pain had fewer deficits on DRS-2 Initiation/Perseveration (12%) and Conceptualization (2%) subscales than patients without pain (31%, *p*=0.028; and 22%, *p*=0.005, respectively) or with non-central parkinsonian pain (28%, *p*=0.039; 22%, *p*=0.004, respectively). No significant differences were found regarding DRS-2 Total score, Attention subscale, Construction subscale, and Memory subscale. Multiple logistic regressions revealed that patients with central parkinsonian pain had lower odds of having deficit on DRS-2 Conceptualization than patients without pain (adjusted odds = 0.08, 95%CI: 0.01, 0.82, *p*=0.034) or patients with non-central parkinsonian pain (adjusted odds = 0.12, 95%CI: 0.01, 1.00, *p*=0.050), when considering age, age at disease onset, agonist medication, and UPDRS-II levodopa responsiveness as covariates. The association between deficit on DRS-2 Initiation/Perseveration and central parkinsonian pain was no longer statistically significant when the same covariates were considered.

The frequency of positive symptoms on QUIP-S was not statistically different between patients with central parkinsonian pain (29.3%) and patients without pain (18.1%) or with non-central parkinsonian pain (25.2%). Though, the frequency of other compulsive behaviors (i.e., hobbyism, punding, and walkabout) was higher in patients with central parkinsonian pain (20%) than in patients with other subtypes of pain (8%, *p*=0.037) and in patients without pain although the level of statistical significance was marginal and nonsignificant (7%, *p*=0.064). The odds of having other compulsive behaviors were higher for patients with central parkinsonian pain than those with non-central pain (adjusted odds = 3.15, 95%CI: 1.00, 9.90, *p*=0.050), when considering age, age at disease onset, agonist medication, and UPDRS-II levodopa responsiveness as covariates. No patient without pain reported compulsive gambling, whereas 7% of patients with central parkinsonian pain or non-central parkinsonian pain had this ICD. No other specific ICDs were related to central parkinsonian pain.

PD patients with central parkinsonian pain (12%) had more current smoking habits than patients without pain (1%, *p*=0.023) or those with non-central parkinsonian pain (3%, *p*=0.043). The odds of having current smoking habits were higher for patients with central pain than patients without pain (adjusted Odds = 6.58, 95%CI: 0.71, 61.38, *p*=0.098), though the level of statistical significance was marginal and nonsignificant when age, age at disease onset, agonist medication, and UPDRS-II levodopa responsiveness were considered. The comparison with patients without pain was no longer statistically significant when the same set of covariates were considered.

## 4. Discussion

The present study revealed that patients with central parkinsonian pain had more compulsive behaviors and addictive habits than patients without pain or those with non-central parkinsonian pain, despite having more preserved cognitive performance.

In our cohort, pain was not related to increased cognitive deficits in the DRS-2. Even though, in non-PD populations, chronic pain has been linked to impairments in memory, attention, and executive functions [16–18] and to an accelerated memory decline and increased probability of dementia [19]; in PD populations, the association between pain and cognitive dysfunction is less clear. There are reports of negative findings [20], but there are also studies that found significant associations between these two nonmotor symptoms in PD [21, 22]. The low sensitivity of DRS-2 to mild deficits and small differences in cognitive functioning can potentially explain the nonsignificant difference between patients without pain and those with non-central parkinsonian pain in our cohort. Interestingly, PD patients with central parkinsonian pain had better cognitive performance in the Initiation/Perseveration and Conceptualization subscales than patients without pain or with non-central parkinsonian pain. These DRS-2 subtests measure executive functions and verbal intelligence [23] and are predictive of dementia in PD [23–25]. This finding suggests that having a more preserved cognition may be a characteristic of the putative Park pain subtype of PD. Noteworthy cognition was assessed under the effects of antiparkinsonian medication. The central parkinsonian pain is believed to be related to a dopaminergic deficit, and there are reports of a greater relief of pain with antiparkinsonian medication than in other types of pain [7, 8].

In our cohort, patients with central parkinsonian pain had more current smoking habits than patients without pain or with non-central parkinsonian pain. However, the frequency of past smoking habits was not different between these groups of PD patients. Epidemiological studies have consistently reported an inverse correlation between tobacco use and PD [26, 27]. There is evidence of a functional interaction between dopamine and nicotinic cholinergic systems and that nicotine may contribute to the symptomatic management of nonmotor symptoms in PD, by stimulating the dopamine release in the striatum [28, 29]. Several studies suggest that nicotine may modulate the nociceptive experience in non-PD patients. So, it is reasonable to speculate that the more frequent current smoking addiction in patients with central parkinsonian pain may be related to a greater and more sustained responsiveness to nicotine.

In our cohort, 5% of all PD patients experienced pathological gambling. This frequency is consistent with the literature. It has been observed that pathological gambling occurs more frequently in PD patients (3.4–6.1%) than in the general population (0.25–2%) [30]. Though, only patients with pain, regardless of the type, reported pathological gambling in our cohort. This finding is consistent with the notion that patients with pain may be more vulnerable to pathological gambling than patients without pain [31, 32]. It has been hypothesized that the inability to cope with painful or uncomfortable physical sensations may drive the gambling behavior, due to a general inability to cope with discomfort. Patients with central parkinsonian pain also reported more other compulsive behaviors (e.g., hobbyism, punding, and walkabout) than patients without pain or with other pain subtypes.

The pathological mechanisms of ICDs in PD are not yet fully understood, but it has been argued that in PD, the dysregulation of two important dopaminergic circuits—the mesolimbic and mesocortical pathways—leads to the clinical manifestation of impulsive and compulsive behaviors [9]. In accordance, neurofunctional studies have found increased functional activation and dopamine release in regions associated with the mesolimbic reward system in PD patients with ICD [33]. In recent years, there has been increasing evidence of the involvement of the mesolimbic system in acute and chronic pain [34]. Chronic pain states may induce changes in neuronal plasticity and functional connectivity in several parts of the brain reward center, including nucleus accumbens, the ventral tegmental area, and the prefrontal cortex [35]. Several studies suggest that the mesolimbic dopamine system modulates the perception of nociceptive information, the efficacy of pain medications, and the affective symptoms of chronic pain [35].

Patients with central parkinsonian pain were younger, took more dopamine agonists, and presented greater responsiveness to levodopa on activities of daily living (as measured by UPDRS-II) than patients without pain or with other types of pain. It can be argued that the behavioral and cognitive features of patients with central parkinsonian pain can be explained, at least in part, by these demographic and clinical characteristics of the patients. In other words, the associations between pain and other nonmotor symptoms may reflect shared protective and risk factors, in addition to possible common pathophysiological mechanisms. Supporting this hypothesis is the reported association between poor quality of sleep and both central parkinsonian pain [8] and ICDs [36] in PD. We observed in the present study that patients with central parkinsonian pain had more preserved cognition. Concurrently, a slower cognitive decline, especially in frontal-lobe-related functions, has been described in PD patients with ICDs [37]. Patients with central parkinsonian pain appear to have a distinct phenotype of PD.

One major strength of this study is the diagnosis of PD and the clinical evaluation by movement disorders specialists, which reduces the risk of misdiagnosis, and the neuropsychological evaluation was performed by an experienced neuropsychologist in the assessment of PD patients. The limitations of the study include the *a priori* exclusion of patients under advanced therapies for PD, namely deep brain stimulation, which reduces the representativeness of the sample, especially in the advanced stages of the disease. The QUIP-S and DRS-2 are recommended by the International Parkinson's and Movement Disorder Society [14] to screen for ICDs and cognitive deficits in PD. These instruments have respectively low specificity and low sensitivity. Not all individuals positive for ICDs on QUIP-S meet the diagnostic criteria, and patients with normal DRS-2 may have cognitive deficits not detected by the instrument.

In summary, patients with central parkinsonian pain are more likely to present certain compulsive and addictive behaviors than patients without pain or those with non-central pain, even though they appear to have more preserved cognition. These findings provide support to the existence of a Park pain phenotype.

## Figures and Tables

**Table 1:** Demographic, clinical, and therapeutic characteristics of Parkinson's disease patients according to pain subtype.

		*A*. PD without pain (*n* = 72)	*B*. PD with non-central pain (*n* = 147)	*C*. PD with central pain (*n* = 41)	*p*		
					*A* vs. *B*	*A* vs. *C*	*B* vs. *C*
Sex—male		44 (61%)	73 (50%)	18 (44%)	0.111	0.077	0.514
Age		72 (64–78)	70 (63–77)	64 (56–71)	0.556	**0.001**	**0.002**
Education		4 (4–9)	4 (4–6)	4 (4–9)	0.254	0.977	0.396
Age at disease onset		63 (55–69)	60 (53–69)	57 (45–63)	0.291	**0.002**	**0.011**
Disease duration (years)		6 (4–13)	8 (4–12)	6 (4–12)	0.030	0.815	0.093
UPDRS-II	OFF	11 (7–17)	15 (9–23)	18 (12–22)	**0.002**	**0.001**	0.274
	ON	6 (3–11)	9 (6–13)	8 (4–11)	**0.002**	0.108	0.360
UPDRS-II levodopa responsiveness		44 (20–63)	36 (19–52)	56 (43–67)	0.156	0.063	**0.001**
UPDRS-III	OFF	30 (22–38)	32 (25–41)	34 (23–42)	0.118	0.122	0.572
	ON	21 (15–26)	21 (16–27)	24 (17–30)	0.481	0.112	0.250
UPDRS-III levodopa responsiveness		32 (22–38)	31 (22–42)	30 (23–42)	0.844	0.766	0.645
L-dopa equivalent (mg)		710 (400–1063)	840 (500–1180)	880 (580–1160)	0.060	0.170	0.974
Agonists		27 (38%)	57 (39%)	24 (59%)	0.855	**0.031**	**0.024**
*Pain treatment*							
	NSAIDs	—	41 (28%)	9 (22%)	—	—	0.447
	Antidepressant	—	4 (3%)	4 (10%)	—	—	0.070
	AEDs	—	6 (4%)	4 (10%)	—	—	0.229
	Paracetamol	—	30 (20%)	11 (27%)	—	—	0.379
	Other drugs	—	14 (10%)	2 (5%)	—	—	0.529
DRS-2	Total	25 (35%)	54 (37%)	11 (27%)	0.771	0.387	0.238
	Attention	21 (29%)	46 (31%)	11 (27%)	0.748	0.791	0.582
	Initiation/Perseveration	22 (31%)	41 (28%)	5 (12%)	0.682	**0.028**	**0.039**
	Construction	21 (29%)	39 (27%)	11 (27%)	0.681	0.791	0.969
	Conceptualization	16 (22%)	32 (22%)	1 (2%)	0.939	**0.005**	**0.004**
	Memory	19 (26%)	36 (25%)	10 (24%)	0.761	0.815	0.990
QUIP-S	Total	13 (18%)	37 (25%)	12 (29%)	0.239	0.167	0.597
	Gambling	0 (0%)	10 (7%)	3 (7%)	**0.033**	**0.046**	>0.999
	Sexual	4 (6%)	15 (10%)	2 (5%)	0.251	>0.999	0.372
	Buying	4 (6%)	5 (3%)	3 (7%)	0.480	0.703	0.375
	Eating	5 (7%)	9 (6%)	1 (2%)	0.777	0.414	0.693
	Other compulsive behaviors	5 (7%)	11 (8%)	8 (20%)	0.886	0.064	**0.037**
	Compulsive medication use	1 (1%)	6 (4%)	0 (0%)	0.287	>0.999	0.342
Smoking	Current habits	1 (1%)	5 (3%)	5 (12%)	0.666	**0.023**	**0.043**
	Past habits	14 (19%)	35 (24%)	10 (24%)	0.451	0.537	0.956

PD: Parkinson's disease; UPDRS: Unified Parkinson's Disease Rating Scale; NSAIDs: nonsteroidal anti-inflammatory drugs; AEDs: antiepileptic drugs; DRS: Dementia Rating Scale; QUIP-S: Impulse Control Disorders in Parkinson's Disease Short Form. Data are presented as frequencies (%) and medians (25^th^–75^th^). Chi-square (or Fisher's exact when appropriate) and Mann–Whitney tests were applied for group comparisons.

## Data Availability

Data not provided in the article will be shared by the authors at the request of other investigators. Database of Centro Hospitalar e Universitário do Porto, Portugal.

## Supplementary Materials

Supplementary Materials Table 1: Clinical classification of painful or unpleasant sensations in PD. Semistructured questionnaire for painis also provided.

## References

[1] Lees A. J., Hardy J., Revesz T. (2009). Parkinson’s disease. *The Lancet*.

[2] Foltynie T., Brayne C., Barker R. A. (2002). The heterogeneity of idiopathic Parkinson’s disease. *Journal of Neurology*.

[3] Sauerbier A., Jenner P., Todorova A., Chaudhuri K. R. (2016). Non motor subtypes and Parkinson’s disease. *Parkinsonism & Related Disorders*.

[4] Marras C., Chaudhuri K. R. (2016). Nonmotor features of Parkinson’s disease subtypes. *Movement Disorders*.

[5] Defazio G., Gigante A., Mancino P., Tinazzi M. (2013). The epidemiology of pain in Parkinson’s disease. *Journal of Neural Transmission*.

[6] Ford B. (2010). Pain in Parkinson’s disease. *Mov Disord*.

[7] Vila-Cha N., Cavaco S., Mendes A. (2019). Unveiling the relationship between central parkinsonian pain and motor symptoms in Parkinson’s disease. *European Journal of Pain*.

[8] Vila-Chã N., Cavaco S., Mendes A. (2019). Sleep disturbances in Parkinson’s disease are associated with central parkinsonian pain. *Journal of Pain Research*.

[9] Latella D., Maggio M. G., Maresca G. (2019). Impulse control disorders in Parkinson’s disease: a systematic review on risk factors and pathophysiology. *Journal of the Neurological Sciences*.

[10] Litvan I., Aarsland D., Adler C. H. (2011). MDS task force on mild cognitive impairment in Parkinson’s disease: critical review of PD-MCI. *Movement Disorders*.

[11] Hughes A. J., Daniel S. E., Kilford L., Lees A. J. (1992). Accuracy of clinical diagnosis of idiopathic Parkinson’s disease: a clinico-pathological study of 100 cases. *Journal of Neurology, Neurosurgery & Psychiatry*.

[12] Fahn S., Elton R., Members of the UPDRS Development Committee, Fahn S M. C., Calne D. B., Lieberman A. (1987). Unified Parkinson’s disease rating scale, recent developments in Parkinson’s disease. *Recent Developments in Parkinson’s Disease*.

[13] Weintraub D., Hoops S., Shea J. A. (2009). Validation of the questionnaire for impulsive-compulsive disorders in Parkinson’s disease. *Movement Disorders*.

[14] Evans A. H., Okai D., Weintraub D. (2019). Scales to assess impulsive and compulsive behaviors in Parkinson’s disease: critique and recommendations. *Movement Disorders*.

[15] Cavaco S T. P. A. (2011). *Manual da Versão Portuguesa da Dementia Rating Scale-2*.

[16] Jarcho J. M., Mayer E. A., Jiang Z. K., Feier N. A., London E. D. (2012). Pain, affective symptoms, and cognitive deficits in patients with cerebral dopamine dysfunction. *Pain*.

[17] Mazza S., Frot M., Rey A. E. (2018). A comprehensive literature review of chronic pain and memory. *Progress in Neuro-Psychopharmacology and Biological Psychiatry*.

[18] Higgins D. M., Martin A. M., Baker D. G., Vasterling J. J., Risbrough V. (2018). The relationship between chronic pain and neurocognitive function. *The Clinical Journal of Pain*.

[19] Whitlock E. L., Diaz-Ramirez L. G., Glymour M. M., Boscardin W. J., Covinsky K. E., Smith A. K. (2017). Association between persistent pain and memory decline and dementia in a longitudinal cohort of elders. *JAMA Internal Medicine*.

[20] Engels G., Weeda W. D., Vlaar A. M. M., Weinstein H. C., Scherder E. J. A. (2016). Clinical pain and neuropsychological functioning in Parkinson’s disease: are they related?. *Parkinson’s Disease*.

[21] Okada A., Nakamura T., Suzuki J. (2016). Impaired pain processing correlates with cognitive impairment in Parkinson’s disease. *Internal Medicine*.

[22] Defazio G., Antonini A., Tinazzi M. (2017). Relationship between pain and motor and non-motor symptoms in Parkinson’s disease. *European Journal of Neurology*.

[23] Brown G. G., Rahill A. A., Gorell J. M. (1999). Validity of the dementia rating scale in assessing cognitive function in Parkinson’s disease. *Journal of Geriatric Psychiatry and Neurology*.

[24] Levy G., Jacobs D. M., Tang M.-X. (2002). Memory and executive function impairment predict dementia in Parkinson’s disease. *Movement Disorders*.

[25] Llebaria G., Pagonabarraga J., Kulisevsky J. (2008). Cut-off score of the mattis dementia rating scale for screening dementia in Parkinson’s disease. *Movement Disorders*.

[26] Ascherio A., Schwarzschild M. A. (2016). The epidemiology of Parkinson’s disease: risk factors and prevention. *The Lancet Neurology*.

[27] Belvisi D., Pellicciari R., Fabbrini A. (2020). Risk factors of Parkinson disease. *Neurology*.

[28] Villafane G., Thiriez C., Audureau E. (2018). High-dose transdermal nicotine in Parkinson’s disease patients: a randomized, open-label, blinded-endpoint evaluation phase 2 study. *European Journal of Neurology*.

[29] Quik M., O’Leary K., Tanner C. M. (2008). Nicotine and Parkinson’s disease: implications for therapy. *Movement Disorders*.

[30] Heiden P., Heinz A., Romanczuk-Seiferth N. (2017). Pathological gambling in Parkinson’s disease: what are the risk factors and what is the role of impulsivity?. *European Journal of Neuroscience*.

[31] Barry D. T., Pilver C. E., Hoff R. A., Potenza M. N. (2013). Pain interference, gambling problem severity, and psychiatric disorders among a nationally representative sample of adults. *Journal of Behavioral Addictions*.

[32] Grant J. E., Chamberlain S. R. (2020). Cold pressor pain and gambling disorder: implications for the opioid system. *CNS Spectrums*.

[33] Steeves T. D., Miyasaki J., Zurowski M. (2009). Increased striatal dopamine release in Parkinsonian patients with pathological gambling: a [11C] raclopride PET study. *Brain*.

[34] Taylor A. M. W., Becker S., Schweinhardt P., Cahill C. (2016). Mesolimbic dopamine signaling in acute and chronic pain. *Pain*.

[35] Mitsi V., Zachariou V. (2016). Modulation of pain, nociception, and analgesia by the brain reward center. *Neuroscience*.

[36] Jesús S., Labrador-Espinosa M. A., Adarmes A. D. (2020). Non-motor symptom burden in patients with Parkinson’s disease with impulse control disorders and compulsive behaviours: results from the COPPADIS cohort. *Scientific Reports*.

[37] Siri C., Cilia R., Reali E. (2015). Long-term cognitive follow-up of Parkinson’s disease patients with impulse control disorders. *Movement Disorders*.

