# Supplementary material for: Central Pain in Parkinson's Disease: Behavioral and Cognitive Characteristics
Source: Parkinsons Dis. 2021 Jun 10;2021:5553460. doi: 10.1155/2021/5553460 (PMC8211520; doi:10.1155/2021/5553460)
Supplement: Supplementary Materials — Table 1: Clinical classification of painful or unpleasant sensations in PD. Semistructured questionnaire for painis also provided. [file 5553460.f1.zip › 5553460.f1/Supplementary material - Semi-structured questionnaire for pain (2).docx]

**Central Pain in Parkinson's disease: behavioral and cognitive characteristics**

**Supplementary material: semi-structured questionnaire for pain**

**Do you have pain?**

Yes __ No ___

(If yes, continue the questionnaire)

**Characteristics**

Burning__ Aching ____ Cramping____ Tingling____ Sense of restlessness___

Other _____________________

**Duration**

<1 month ___ 1 - 3 months ___ 3 months - 1 year ___ More than 1 year____

**Age of onset** ___

**Localization** ____

**Frequency**

Continuous (always/every day) ___

Recurrent (several days a week to several days a month) ___

Sporadic (less than a few days a month) __

**Intensity**

Mild___ Moderate___ Intense ____

**Aggravating Factors**

Movement ___ Rest ____ Temperature change ___

Other ______________________________________

**Relief Factors**

Movement ___ Rest ____ Temperature change ___

Other ______________________________________

**Drugs Dosis**

Amitriptyline _______

Gabapentin ________

Pergabalin ________

Venlafaxine ________

Paracetamol ________

NSAIDs (______) ________

COX inhibitor ________

Tramadol ________

Other (____) _____

**Relief with medication**

0% 10% 20% 30% 40% 50% 60% 70% 80% 90% 100%

**Alternative Therapeutics**

No_ Acupuncture__ Massages__ Meditation___ "Natural" Pharmacies ___ Other ____

**Relief with alternative therapeutics**

0% 10% 20% 30% 40% 50% 60% 70% 80% 90% 100%

**Satisfaction with pain management**

Unsatisfied __ Slightly Satisfied __ Moderately satisfied __ Very satisfied ___

**Relationship with Parkinson's Disease**

Do you think it is related to PD? No____ Yes_____

Before start of PD_______ After start of PD ________

Topographical relation to PD? No____ Yes_____

Relation to dystonia? No ____ Yes _____

Does it improve with anti-parkinsonian medication? No ____ Yes_____

Relation with dyskinesias? No ____ Yes __

Does it worsen with anti-parkinsonian medication? No ____ Yes _____

Does the pain have fluctuations? No ____ Yes _____

What time of day do you have the most pain?

Morning ______ Afternoon _______ Night ___ Early morning ____

**Neurological exam**

Motor or sensory signs of nerve or root entrapment? Yes ___ No___

Changes other than extrapyramidal signs ______________________

**Classification of pain (Table 1)**
