# Supplementary material for: Central Pain in Parkinson's Disease: Behavioral and Cognitive Characteristics
Source: Parkinsons Dis. 2021 Jun 10;2021:5553460. doi: 10.1155/2021/5553460 (PMC8211520; doi:10.1155/2021/5553460)
Supplement: Supplementary Materials — Table 1: Clinical classification of painful or unpleasant sensations in PD. Semistructured questionnaire for painis also provided. [file 5553460.f1.zip › 5553460.f1/Supplementary material Table 1.docx]

**Supplementary material – Table 1**

Table 1. *Clinical classification of painful or unpleasant sensations in PD*

| **Category by description** | **Clinical features** | **Check** |
| --- | --- | --- |
| **Musculoskeletal** | Aching, cramping, arthralgic, myalgic sensations in joints, and muscles;  Associated findings may include muscle tenderness, arthritic changes, skeletal deformity, limited joint mobility, postural abnormalities, and antalgic gait;  May be exacerbated by parkinsonian rigidity, stiffness, and immobility, and relieved by mobility;  May fluctuate with medication dosing, and improve with levodopa |  |
| **Dystonic** | Associated with sustained twisting movements and postures;  Muscular contractions often very forceful and painful;  Dystonia may involve any limb or extremity, as well as facial and pharyngeal musculature;  May fluctuate closely with medication dosing: early morning dystonia, off dystonia, beginning-of-dose and end-of-dose dystonia, peak dose dystonia |  |
| **Radicular/neuropathic** | Pain in a root or nerve territory, associated with motor or sensory signs of nerve or root entrapment |  |
| **Central parkinsonian pain** | Burning, tingling, formication, ‘‘neuropathic’’ sensations, often relentless and bizarre in quality, not confined to root or nerve territory;  Pain may have an autonomic character, with visceral sensations or dyspnea, and vary in parallel with the medication cycle as a non-motor fluctuation.  Not explained by rigidity, dystonia, musculoskeletal or internal lesion |  |
| **Akathisia** | Subjective sense of restlessness, often accompanied by an urge to move.  May fluctuate with medication effect, and improve with levodopa |  |

From: Ford, B., *Pain in Parkinson's disease.* Mov Disord, 2010. 25 Suppl 1: p. S98-103.
